# A community based cross sectional study on the prevalence of dyslipidemias and 10 years cardiovascular risk scores in adults in Asmara, Eritrea

**DOI:** 10.1038/s41598-022-09446-9

**Published:** 2022-04-02

**Authors:** Oliver Okoth Achila, Nahom Fessahye, Samuel Tekle Mengistu, Naemi Tesfamariam Habtemikael, Wintana Yebio Werke, Femal Tesfazghi Zemichael, Haben Negash Leghese, Thomas Amanuel Weldegegish, Tsegay Habteab Tekeste, Eyob Yohannes Garoy

**Affiliations:** 1 Orotta College of Medicine and Health Sciences, Asmara, Eritrea; 2 Nakfa Hospital, Ministry of Health Northern Red Sea Branch, Nakfa, Eritrea

**Keywords:** Lipids, Cardiology, Cardiovascular diseases, Metabolic disorders

## Abstract

Despite the contribution of dyslipidemia to the high and rising burden of arteriosclerotic cardiovascular disease (CVD) in Sub-Saharan Africa; the condition is under-diagnosed, under-treated, and under-described. The objective of this study was to explore the prevalence of dyslipidemias, estimate a 10-year cardiovascular disease risk and associated factors in adults (≥ 35 to ≤ 85 years) living in Asmara, Eritrea. This population-based cross-sectional study was conducted among individuals without overt CVDs in Asmara, Eritrea, from October 2020 to November 2020. After stratified multistage sampling, a total of 386 (144 (37%) males and 242 (63%) females, mean age ± SD, 52.17 ± 13.29 years) respondents were randomly selected. The WHO NCD STEPS instrument version 3.1 questionnaire was used to collect data. Information on socio-demographic variables was collected via interviews by trained data collectors. Measurements/or analyses including anthropometric, lipid panel, fasting plasma glucose, and blood pressure were also undertaken. Finally, data was analyzed by using Statistical Package for Social Sciences version 26.0 for Windows (SPSS Inc., Chicago, IL, USA). All *p*-values were 2-sided and the level of significance was set at *p* < 0.05 for all analyses. The frequency of dyslipidemia in this population was disproportionately high (87.4%) with the worst affected subgroup in the 51–60 age band. Further, 98% of the study participants were not aware of their diagnosis. In terms of individual lipid markers, the proportions were as follows: low HDL-C (55.2%); high TC (49.7%); high LDL (44.8%); high TG (38.1%). The mean ± SD, for HDL-C, TC, LDL-C, non-HDL-C, and TG were 45.28 ± 9.60; 205.24 ± 45.77; 130.77 ± 36.15; 160.22 ± 42.09 and 144.5 ± 61.26 mg/dL, respectively. Regarding NCEP ATP III risk criteria, 17.6%, 19.4%, 16.3%, 19.7%, and 54.7% were in high or very high-risk categories for TC, Non-HDL-C, TG, LDL-C, and HDL-C, respectively. Among all respondents, 59.6% had mixed dyslipidemias with TC + TG + LDL-C dominating. In addition, 27.3%, 28.04%, 23.0%, and 8.6% had abnormalities in 1, 2, 3 and 4 lipid abnormalities, respectively. Multivariate logistic regression modeling suggested that dyslipidemia was lower in subjects who were employed (aOR 0.48, 95% CI 0.24–0.97, *p* = 0.015); self-employed (aOR 0.41, 95% CI 0.17–1.00, *p* = 0.018); and married (aOR 2.35, 95% CI 1.19–4.66, *p* = 0.009). A higher likelihood of dyslipidemia was also associated with increasing DBP (aOR 1.04 mmHg (1.00–1.09, *p* = 0.001) and increasing FPG (aOR 1.02 per 1 mg/dL, 95% CI 1.00–1.05, *p* = 0.001). Separately, Framingham CVD Risk score estimates suggested that 12.7% and 2.8% were at 10 years CVD high risk or very high-risk strata. High frequency of poor lipid health may be a prominent contributor to the high burden of atherosclerotic CVDs—related mortality and morbidity in Asmara, Eritrea. Consequently, efforts directed at early detection, and evidence-based interventions are warranted. The low awareness rate also points at education within the population as a possible intervention pathway.

## Introduction

Cardiovascular diseases (CVDs) are a leading cause of mortality and morbidity worldwide. Apart from the high morbimortality, the disease is associated with increases in years of life lost (YLLs), years lived with disability (YLDs), and disability-adjusted life-years (DALY)^1,2^. To illustrate, the 2013 global burden of disease (GBD) estimate suggested that CVD caused ~ 17.8 million deaths globally, corresponding to 330 million YLLs and another 35.6 million years YLDs^3^. In 2019, ischaemic heart disease (IHD) and cerebrovascular disease (stroke) were the top-ranked causes of DALYs in persons above 50 years^2,3^. A notable aspect of the ongoing carnage is that low and medium-income countries (LMIC) are disproportionately impacted^4^. For example, data suggest that sub-Saharan Africa (SSA) bears the highest burden of stroke globally (age-standardized stroke incidence rates of ~ 316 per 100,000)^5,6^. In absolute numbers, CVD causes four to five times as many deaths in LMICs as in high-income countries (HICs)^7^. On the current trajectory, the 2030 UN Sustainable Development Goals (SDGs) (adopted in 2015) to reduce premature deaths from NCDs by a third in Africa are unlikely to be achieved^7^.

The disproportionate impact of CVD in LMIC is largely driven by a complex interplay between population-wide changes in socio-demographic, economic, and lifestyle factors^6,7^. Another well-established, most common, and readily detectable and treatable driver of CVD in SSA is dyslipidemia (Total cholesterol (TC) ≥ 200 mg/dL, Triglycerides ≥ 150 mg/dL, LDL-C ≥ 130 mg/dL, HDL-C ≤ 40 mg/dL; male, HDL-C ≤ 50 mg/dL; female)^4,6^. Globally GBD project estimated that TC causes about 18% of strokes and 56% of ischemic heart disease (IHD), accounting for 4.4 million deaths annually and 93.8 million DALYs^2^. More importantly, the highly regarded INTERHEART study demonstrated that dyslipidemia is one of the leading population-level risk factors for Ischemic Heart Disease (IHD) in SSA^8^. This, therefore, cannot be overemphasized: poor lipid health in adult populations in SSA is emerging as a true epidemic.

Despite the contribution of dyslipidemia to the high and rising burden of CVD in SSA^4,6^; the condition is under-diagnosed, under-treated and, under-described. Although national guidelines, such as those from the American College of Cardiology/American Heart Association (ACC/AHA); the US National Cholesterol Education Program Adult Treatment Panel III (NCEP-ATP III); and the American Association of Clinical Endocrinologists (AACE) have emphasized the need for early screening for lipid abnormalities^9–11^; the practice is rare in SSA. This represents a missed opportunity given the fact that targeting risk drivers of CVD at the population level by a combination of simple, low cost, approaches could avert more than 50% of the attributable morbidity and mortality^12^. Previously identified barriers to addressing this problem include lack of awareness (among the public and health-care professionals); high cost of diagnosis and treatment; lack of local clinical practice guidelines; under-treatment and a limited understanding of its epidemiology^4^. The lack of reliable health information/statistics and a severe lack of community-based epidemiological data is a source of serious concern as it handicaps public health strategies directed at prevention and treatment/or management. Significantly, the shortage of historical data limits reliable assessment of trends.

Much of the description in the foregoing paragraph applies to Eritrea. The lack of data is extremely concerning given the fact that World Health Organization (WHO) fact-sheets have consistently shown that CVD-related mortality in the country is disproportionately high (388.1 vs. 282.2 per 100 000 in males and females, respectively)^13^. Interestingly, the country has one of the lowest prevalence of overweight/obesity (mean BMI = 20.5 (95% CI 19.9–21.1 and BMI ≥ 25 kg/m^2^ = 17.7% (95% CI 14.7–20.2%) in SSA^13,14^. The prevalence of other known drivers such as tobacco use, irresponsible alcohol consumption, hypertension, and Diabetes mellitus are modest or low^3^. Importantly, healthy life expectancy (HALE) increased from 30.7 years [28.9–32.2] in 1990 to 54.4 years [51.5–57.1] in 2017)^2^. One facet of this problem that has received little attention is the burden of dyslipidemia and its possible contribution to the excess burden of CVD in the country.

Therefore, the primary objective of this study was to generate population-level data on the burden of dyslipidemia in the adult population in Asmara, Eritrea. Beyond the focus on dyslipidemia, using multivariable risk scores/prediction algorithms to identify persons at higher risk is a well-established intervention strategy^15,16^. To this end, we computed 10-year general CVD risk scores using the Framingham risk score calculator. Information from Framingham CVD Risk estimates can be crucial in designing evidence-based, context-specific community-level and/or individualized interventions. The data can also be leveraged in the future to evaluate trends associated with major CVDs risk factors. Importantly, estimating CVD risk using the Framingham CVD Risk Score presupposes the collection of data on an expanded list of risk factors. Therefore, and as a secondary objective, data regarding the prevalence and distribution of individual risk factors such as obesity, fasting blood sugar (FPG), blood pressure (BP), among others were collected.

## Methods and design

### Study design, setting, and participants

This population-based cross-sectional study was conducted in Asmara, Eritrea, from October 2020 to November 2020. Individuals aged between 35 and 75 years living in the study area were targeted. Located in the central region (Zoba Maekel), Asmara serves as the capital city and has the largest population cluster in the country (approximately 658,516 persons). Administratively, the city is divided into 13 *Zobas* (sub-zones) (Mai-Temenay, Edaga-Hamus, Akria, Paradizo, Aba-Shawel, Arbaete-Asmara, Maekel-Ketema, Tsetserat, Tiravelo, Sembel, Godaef, Gejeret and Geza-banda).

### Sample size determination and sampling procedure

“A single population-proportion formula was used to determine the sample size^17^.” Using a proportion of dyslipidemia to be 70% (we used 70% prevalence)^18^, 95% confidence interval (CI), 5% type I error level, 80% power, and design effect of 1.2; the sample size was calculated to be close to 386 participants.

**Figure 1:**
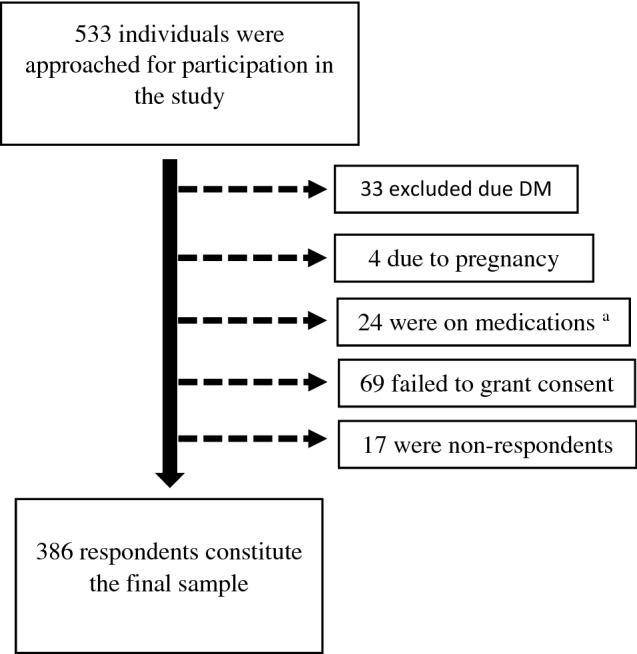
Flow chart for study participant recruitment. ^a^Steroid, β-adrenergic blockers, thiazide diuretics, anti-IV medication, and/or statin.

To identify eligible study participants, a stratified multistage sampling design was followed. Briefly, the total sample size was proportionally allocated to the 13 sub-zones. After eliminating sparsely populated Zip-codes within each Sub-zone, one Zipcode (enumeration area (EA)) was selected using a simple random sampling technique (computer-based random number generator was applied) within each sub-zone. The appropriate number of households (HH) per EA was subsequently selected using random sampling. Households were excluded from the study if all members of the HH were outside the required age range (≥ 35 to ≤ 85 years). If an abandoned house was encountered during the random selection, it was replaced by the next inhabited household. Eligible participants per HH were selected using the Kish method (a random selection of eligible individuals at the HH level)—eligible members of each household were assigned numbers (starting with the youngest). The Kish grid was then used to identify the participant. In the absence of eligible participants during the visit, a second visit was offered to grant potential participants the opportunity to participate in the survey. In case of the participant’s absence during the entire study period, replacement by the current person who was 35 and above was undertaken. All eligible individuals who provided written informed consent were enrolled. Ultimately, we included individuals who met the following criteria: willingness to grant consent, age ≥ 35–85 years, and had permanently resided in Asmara, Eritrea for at least one year. Exclusion criteria included the following: bedridden subjects, pregnancy, serious mental disorder, hearing or intellectual disability, breastfeeding mothers, individuals who were unwilling to provide consent, individuals on specific medications (Steroid, β-adrenergic blockers, thiazide diuretics, anti-HIV medication, statins, among others); and individuals with Diabetes Mellitus (DM). In all, 533 individuals were approached for participation in the study. See Fig. 1.

### Data collection, measurements, and definitions

Data were collected using a modified version of the WHO NCD STEPS instrument version 3.1^19^. To accommodate unlettered participants, the questionnaire was translated from English to Tigrigna (a local Language) by a language expert. And it was administered to the chosen individuals by trained data collectors. Overall, the instrument incorporates queries on a range of well-established cardio-metabolic risk factors and is separated into four sections/Steps. Step 1, includes questions on socio-demographic characteristics (sex, age, the highest level of education, occupation, marital status, ethnicity as well as family history of DM (diabetes mellitus); Step 2 explores lifestyle factors (exercising, sedentary lifestyle, smoking, alcohol consumption, and history of hypertension); Step 3 explores physical measurement (anthropomorphic measurements, blood pressure (BP) measurements, among others); and Step 4 describes biochemical measurements including Fasting plasma glucose (FPG), TG, TC, HDL.

### Anthropometry and blood pressure measurement

#### Anthropometric measurements

Standardized techniques following WHO-STEPS surveillance manual and calibrated equipments were used for anthropometric measurements. All anthropometric measurements were performed by well-trained investigators. Weight, Height, Hip circumference (measured at the widest part of the buttocks), and WC (measured at the iliac crest) were measured as per established protocols using standardized instruments/equipments—a constant tension tape and pre-calibrated digital weighing scale (Sunbeam EB710 digital bathroom scale).

Abdominal obesity was defined as per International Diabetes Federation (IDF) specification (WC ≥ 94 cm in males and ≥ 80 cm in females)^20^. For population-level comparisons, overweight and obesity were defined using body mass index (BMI)—(where BMI = weight in Kilogram (kg)/Height in meters (m)^2^. A per the WHO BMI specification, a BMI < 18.5 kg/m^2^ was categorized as underweight; ≥ 18.5–24.9 kg/m^2^ was classified as normal weight; BMI ≥ 25–29.9 kg/m^2^ as being overweight and a BMI ≥ 30 kg/m^2^ was classified as obese. The waist-to-hip ratio (WHR) and the waist-to-height ratio (WHtR) were also calculated. For purposes of analysis, a WHR ≥ 0.90 for men and ≥ 0.85 for women were considered abnormal as per IDF guidelines^20^.

#### Blood pressure (BP)

Sitting BP was measured as per 1999 WHO/International Society of Hypertension guidelines for the management of hypertension protocol^21^, on the first day of contact, using a standard adult arm cuff of a well-calibrated Omron Digital Blood Pressure machine (OMRON HEM-705 brand PC, Tokyo Japan). After 10 min of rest, 3 measurements were taken after an interval of ~ 5 min. The participant BP was computed from the average of the 2nd and 3rd measurements. Systemic hypertension (HTN) was defined as systolic blood pressure (SBP) ≥ 140 mmHg or a diastolic blood pressure (DBP) ≥ 90 mmHg^22^, or previous diagnosis of HTN or self-reports of antihypertensive medication use.

### Ethics approval and consent to participate

Ethical approval for the study and experimental protocols used was obtained from Eritrean Ministry of Health (MOH) research ethical committee. Informed consent was obtained from all participants. During the study, strict adherence to approved laboratory protocols was observed. This study conforms to the principles outlined in the Declaration of Helsinki.

## Biochemical testing

### Specimen collection and analysis

Participants who consented to participate in the study were requested to fast overnight (from 9 P.M.) before blood sample collection between 8 and 9 A.M. As per established protocols, 5 ml of blood was obtained from the median cubital vein, after > 9 h of fasting. The samples were transported in an icebox within 2 h of collection for processing at Sembel Hospital clinical chemistry laboratory. Total Cholesterol (TC), Triacylglycerol (TG), and High-density lipoprotein cholesterol (HDL-C), Fasting Plasma Glucose (FPG) were analyzed, as per manufacturer instructional guidelines, using Beckman Coulter (AU480 Chemistry System). In addition, quality control measures were performed according to the manufacturer’s recommendation and laboratory guidelines.

#### Lipid panel

As highlighted above, TC, TG, HDL-C were evaluated. The lipid panel components were categorized using the National Cholesterol Education Program Adult Treatment Panel III (NCEP-ATP III) and ADA guidelines^9^. Separately, Friedewald formula (LDL [mg/dL] = total cholesterol [mg/dL]—HDL [mg/dL]—triglycerides [mg/dL]/5) was used to estimate LDL-C concentration (participants with TG level > 400 mg/dL were excluded in this analysis). In addition, non-HDL-C was computed as TC-HDL-C. We calculated four lipid ratios: TG/HDL; TC/HDL; LDL/HDL and non-HDL/HDL ratio. A TG/HDL ratio ≥ 5 was regarded as abnormal.

Dyslipidaemia was defined as any of the following abnormalities: TC ≥ 200 mg/dL (≥ 5.2 mmol/L); LDL-C ≥ 130 mg/dL (≥ 3.4 mmol/L); TG ≥ 150 mg/dL (≥ 1.7 mmol/L); HDL-C (≤ 40 mg/dL (< 1.04 mmol/L) in male and ≤ 50 mg/dL (< 1.3 mmol/L) in female)^9^ or reported use of anti-lipid medication. Further, mixed-dyslipidemia was defined as the concurrent presence of 2 or more lipoprotein abnormalities.

#### Fasting plasma glucose and Glycated hemoglobin (HbA_1C_)

FPG was analyzed in all consenting patients. Accordingly, the enrollees were classified as normal (FPG < 100 mg/dL) (< 5.6 mmol/L); Prediabetes (FPG ≥ 100 and ≤ 125 mg/dL) (< 5.6–≤ 6.9 mmol/L); undiagnosed DM (FPG ≥ 125 mg/dL) (≥ 7 mmol/L)^20^. Taking into account the cost of the study and the level of sensitivity and specificity required for presumptive diagnosis of DM, HbA1c analysis was restricted to FPG ≥ 125 mg/dL. As per ADA criteria, HbA_1C_ < 5.7% was regarded as normal; HbA_1C_ between 5.7 and 6.4% was classified as pre-diabetes and HbA_1C_ > 6.5% was classified as undiagnosed DM.

### Framingham CVD risk score

The Framingham CVD Risk Score Calculator (10-years general cardiovascular disease: Framingham, 2008 paper) was used to estimate 10-year CVD risk. The calculator incorporates a range of traditional CVD risk markers including HDL, age range, hypertension treatment, smoking, and TC. Similar, to Reiger et al., Framingham CVD Risk Score was ascribed to participants based upon the low risk (< 3%); moderate (≤ 3–< 15%); high (≥ 15–< 30%), and very high (≥ 30%)^23^.

#### Data analysis

The completed questionnaires were entered on CSPro software (version 7.0). Keying errors were handled by the double-entry of data. The data was analyzed using Statistical Package for Social Sciences version 20.0 for Windows (SPSS Inc., Chicago, IL, USA). Enrollee characteristics were summarized using frequencies and percentages. Depending on the distribution, continuous data were presented as mean ± standard deviation (SD) or median ± interquartile range (IQR). Data normality, homogeneity of variance, and multicollinearity were tested using suitable statistics. Unadjusted statistical comparisons between categorical variables and categorical outcomes were made using the Chi-square (*χ*^2^) test or Fisher exact test. Depending on data distribution, the *t*-test and one-way analysis of variance (ANOVA) or their non-parametric equivalents (Mann–Whitney *U* tests or Kruskal Wallis) were employed. Multivariable logistic regression models (backward: conditional) were fitted to identify independent predictors of elevated TC, TG, LDL-C, non-HDL-C, low HDL, and dyslipidemia. Subsequently, crude (COR) and adjusted odds ratios (aOR) and associated 95% confidence (95% CI) were reported. To correct for the impact of multiple comparisons, the Bonferroni correction was applied. All *p*-values were 2-sided and the level of significance was set at *p* < 0.05 for all analyses. Missing values or refusals to answer questions were handled by exclusion from analysis.

### Ethical consideration

Administrative and ethical approval was granted by the Eritrean Ministry of Health (EMOH) research proposal review and ethical clearance committee. Written informed consent was obtained from each participant in the local language (Tigrigna) as per the procedures approved by the EMOH ethical committee. Importantly, enrollees were duly informed of their non-negotiable right to instantly terminate their participation in the study. Strict adherence to approved laboratory protocols was observed during specimen collection, processing, and testing. All methods were performed in accordance with the national guidelines and regulations.

## Results

### Demographic characteristics, patient history, anthropometry, and clinical measurements

Table 1 presents the general characteristics of the 386 participants eligible for this study. In general, 144 (37.5%) respondents were males and 242 (63%) were females. The mean ± SD age of the respondents was 52.17 ± 13.29 years (male, 54.85 ± 14.81 vs. female, 50.57 ± 12.04 years; *p* value = 0.004). The BMI ranged from 15.81 to 39.56 kg/m^2^, with a mean of 24.82 ± 4.09 kg/m^2^ (95% CI 24.4–25.2). Females compared to males had a lower level of education (No formal education: 79.2% in females); more likely to be single (62.5%), divorced (84.6%), or widowed (84.6%); more likely to be unemployed (88.1%) and were less likely to smoke (15.41%). Further, 98% of the study participants were not aware that they had a particular abnormality in any lipid marker. See Table 1 for additional information.

**Table 1:** Demographic characteristics, patient history, anthropometry, and clinical measurements at inclusion in the study.

Variables	Male No. (%)	Female No. (%)	*P*-value (*χ*^2^)	Total No. (%)
**Age (years)**				
≤ 40	27 (32.2)	57 (67.9)	0.132 (5.607)	84 (21.8)
40–50	43 (33.6)	85 (66.4)		128 (33.2)
51–59	28 (36.8)	48 (63.2)		76 (19.7)
≥ 60	46 (46.9)	52 (21.5)		98 (25.4)
**Educational level**				
No formal **education/elementary**	16 (20.8)	61 (79.2)	**< 0.001 (41.46)**	77 (19.9)
Junior	27 (34.2)	52 (65.8)		79 (20.5)
High school	47 (31.5)	102 (68.5)		149 (38.6)
Higher Education	54 (66.7)	27 (33.3)		81 (21.0)
**Marital status**				
Single	12 (37.5)	20 (62.5)	**< 0.001 (12.50)**	32 (8.3)
Married	124 (41.1)	178 (58.9)		302 (78.2)
Divorced	2 (15.4)	11 (84.6)		13 (3.4)
Widowed	6 (15.4)	33 (84.6)		39 (10.1)
**Occupation**				
Unemployed	24 (11,9)	178 (88.1)	**< 0.001 (162.46)**	202 (52.33)
Self-employed	40 (72.7)	15 (27.3)		55 (14.24)
Government employee	65 (45.1)	36 (14.9)		101 (26.16)
Privately employed	15 (53.6)	13 (46.4)		28 (7.25)
**Smoking history**				
Yes	11 (84.6)	2 (015.41)	**0.001 (12.87)**	13 (3.4)
No	133 (35.7)	240 (64.3)		373 (96.6)
**Alcohol consumption**				
Yes	101 (40.7)	147 (59.3)	0.079 (3.50)	248 (64.2)
No	43 (31.2)	95 (68.8)		138 (35.8)
**Previous diagnosis of hypertension**				
Yes	25 (36.2)	44 (63.8)	0.041 (0.891)	69 (17.9)
No	119 (37.5)	198 (62.5)		317 (82.1)
**BP (mmHg)**				
SBP > 1**30**	48 (39.3)	74 (60.7)	0.317 (0.574)	122 (31.6)
DBP > 85	45 (42.5)	61 (57.5)	0.238 (1.66)	106 (27.5)
Elevated BP (> 140/90)	35 (37.2)	59 (62.8)	0.544 (0.000)	94 (24.4)
**WC (abnormal)**	67 (24.2)	210 (75.80)	**< 0.001 (72.17)**	277 (71.8)
**WHR (≥ 0.95 (men)/ ≥ 0.80 (women)**				
**WHtR (> 0.50)**	115 (79.9)	218 (90.1%)	0.006 (7.96)	0.57 ± 0.7
**BMI (kg/m**^**2**^**)**				**24.82 ± 4.01**
< 18.5	4 (23.5)	13 (76.5)	**< 0.001 (19.33)**	17 (4.4)
18.5–24.9	91 (46.2)	106 (53.8)		197 (51.0)
25–29.9	43 (33.6)	85 (66.4)		128 (33.2)
> 30	6 (13.6)	38 (86.4)		44 (11.4)
**FPG (mg/dL)**				**96.17 ± 22.96**
Normal < 100	106 (36.7)	183 (63.3)	0.599 (1.025)	289 (75.3)
IFP (≥ 100–125)	31 (41.3)	44 (58.7)		75 (19.5)
DM (> 125)	6 (30.0)	14 (70.0)		20 (5.2)

Significant values are in bold. *P* values (2 tailed): Frequencies of specific demographic and clinical variables between males and females and associated Chi squire/ Fishers exact test values or student *t* test values.

*BP* blood pressure, *SBP* systolic blood pressure, *DBP* diastolic blood pressure, *WC* waist circumference, *WHR* waist/Hip ratio, *WHR* waist/height ratio, *BMI* body mass index, *FPG* fasting plasma glucose.

### Relationship between specific demographic characteristics, anthropometry, clinical measurements, and specific lipid markers

The relationship between specific demographic characteristics, anthropometry, clinical measurements, and specific lipid markers was also evaluated. Overall, women had significantly higher TC (209.9 ± 49.32 vs. 197.39 ± 37.96 mg/dL, *p* = 0.009), HDL-C (47.53 ± 9.7 vs. 41.5 ± 8.09 mg/dL, *p* = 0.02) and LDL-C (133.98 ± 38.31 vs. 125.35 ± 31.56 mg/dL, *p* = 0.02) compared to men. In contrast, men had higher mean values for TGs (153.5 ± 62.59 vs. 139 ± 59 mg/dL, *p* = 0.026) and TC/HDL ratio (4.84 ± 0.91 vs. 4.50 ± 1.00, *p* < 0.001). In addition, respondents with prediabetes and undiagnosed DM had higher values in TG compared to respondents with normal FPG; lower HDL-C, and higher TC/HDL ratio. Finally, increasing age (to ≤ 60 years) was characterized by higher values in TC, LDL-C, non-HDL-C, and TC/HDL ratio. See Table 2 for additional information.

**Table 2:** Relationship between specific demographic characteristics, anthropometry, clinical measurements and specific lipid markers.

Variables	TC mg/dL (Mean ± SD)	TG mg/dL (Mean ± SD)	HDL-C mg/dL (Mean ± SD)	LDL-C mg/dL (Mean ± SD)	Non-HDL-C mg/dL (Mean ± SD)	TC/HDL-C (Mean ± SD)
**Gender**						
Male	197.39 ± 37.96	153.5 ± 62.59	41.5 ± 8.09	125.35 ± 31.56	155.89 ± 34.62	4.84 ± 0.91
Female	209.9 ± 49.32	139 ± 59.9	47.53 ± 9.7	133.98 ± 38.31	162.79 ± 45.83	4.50 ± 1.00
*p*-value for difference	**0.009**	**0.026**	**< 0.001**	**0.02**	0.119	**0.001**
**Age (years)**						
< 40 years	191.58 ± 36.2	131 ± 57.97	44.8 ± 7.37	120.46 ± 29.9	146.77 ± 34.40	4.34 ± 0.87
40–50 years	205.89 ± 49.6	146.48 ± 67.0	44.95 ± 10.18	130.12 ± 35.6	161.32 ± 45.04	4.67 ± 0.95
51–60 years	213.9 ± 40.67	154.7 ± 60.58	45.25 ± 9.9	137.7 ± 33.17	168.67 ± 37.26	4.86 ± 1.12
> 60 years	209.3 ± 49.5	145.3 ± 54.9	46.1 ± 10.28	135 ± 41.9	163.74 ± 45.41	4.63 ± 0.98
*p*-value for difference	**0.01**	0.100	0.760	**0.011**	**0.006**	**0.009**
**Educational level**						
No formal education	210.48 ± 48.54	136.1 ± 40.14	46.5 ± 10.9	137 ± 45.2	165.55 ± 46.75	4.62 ± 1.04
Elementary	198 ± 46.9	140.2 ± 58.1	46.4 ± 9.8	124.4 ± 37.36	151.59 ± 43.06	4.34 ± 0.93
Junior	211 ± 49.1	148.6 ± 62.5	45.9 ± 8.8	132.68 ± 31.78	165.13 ± 45.49	4.67 ± 0.97
Secondary	208.6 ± 44.0	145.41 ± 63.9	45.7 ± 10.0	134.4 ± 37.1	162.95 ± 40.21	4.68 ± 1.03
Tertiary	195 ± 42.6	144.2 ± 64.2	42.7 ± 8.37	122.94 ± 32.5	152.40 ± 38.57	4.64 ± 0.91
*p*-value for difference	0.100	0.88	0.100	0.099	0.142	0.359
**Marital status**						
Married	204.81 ± 44.5	145.95 ± 63.0	44.58 ± 9.37	130.5 ± 33.8	160.38 ± 40.69	4.68 ± 0.98
Single	201.8 ± 53.7	137.6 ± 51.9	46.2 ± 10	128.1 ± 45	155.56 ± 47.39	4.40 ± 0.82
Divorced	195.8 ± 34.8	129.1 ± 64.5	47.6 ± 13.15	122.4 ± 32.69	148.23 ± 32.24	4.33 ± 1.18
Widowed)	214.46 ± 51	144.25 ± 53.3	49.1 ± 8.8	137.38 ± 45.9	166.74 ± 50.57	4.44 ± 1.05
*p*-value for difference	0.500	0.700	0.03	0.54	0.497	0.147
**Alcohol consumption**						
No	196.6 ± 49.4	135.02 ± 56.64	44.05 ± 9.65	133.67 ± 35.04	152.59 ± 45.98	4.56 ± 1.12
Yes	210.0 ± 42.97	149.60 ± 63.14	45.97 ± 9.51	125.7 ± 37.63	162.46 ± 39.21	4.66 ± 0.90
*p-*value for difference	**0.006**	**0.023**	0.061	**0.042**	**0.011**	0.401
**Waist circumference (cm)**						
Normal	191.79 ± 39.7	138.28 ± 61	43 ± 8.88	122.19 ± 33.9	148.77 ± 37.64	4.57 ± 1.06
Increased*	210.5 ± 46.9	146.9 ± 61.29	46.1 ± 9.7	134.15 ± 36.48	164.72 ± 42.95	4.65 ± 0.95
*p*-value for difference	** < 0.001**	0.200	**0.004**	**0.003**	**0.001**	0.524
**BMI (kg/m**^**2**^**)**						
< 18.5	186.6 ± 42	95.6 ± 39.7	47.88 ± 10.9	119 ± 30.45	138.76 ± 37.78	3.99 ± 0.95
18.5–24.9	205.3 ± 46.5	140.8 ± 59.8	45.3 ± 9.09	131.7 ± 36.17	160.28 ± 44.09	4.63 ± 1.08
25–29.9	206.3 ± 47	154.36 ± 64.7	44.89 ± 10.7	129.9 ± 37.44	161.82 ± 40.81	4.68 ± 0.84
> 30	208.8 ± 39	149.5 ± 54.7	45.29 ± 7.79	133.6 ± 34.38	163.55 ± 36.69	4.68 ± 0.88
*p*-value for difference	0.369	**0.002**	0.69	0.51	0.181	0.055
**WHR**						
Normal	195.3 ± 45.12	128.4 ± 53.1	46.1 ± 10.59	124.9 ± 37.9	149.24 ± 40.54	4.34 ± 1.02
Abnormal	207.3 ± 45.69	147.9 ± 62.4	45.1 ± 9.37	132 ± 35.7	162.57 ± 42.10	4.68 ± 0.97
*p*-value for difference	**0.049**	**0.018**	0.430	0.140	**0.016**	**0.012**
**Fasting plasma glucose (mg/dL)**						
< 100	203.2 ± 45.9	140.35 ± 61.15	45.99 ± 9.4	128.82 ± 34.8	157.37 ± 42.65	4.49 ± 0.92
100–124.9	210.56 ± 45.3	152.3 ± 57.5	43.48 ± 10.27	136.35 ± 39.05	167.57 ± 39.67	4.96 ± 1.07
> 125	214.95 ± 44.3	173.35 ± 68.4	41.6 ± 8.32	138.68 ± 42.17	173.35 ± 40.45	5.25 ± 1.01
***p*-value for difference**	0.290	0.03	0.027	0.170	0.063	0.001
**Total**	**205.24 ± 45.77**	**144.5 ± 61.26**	**45.28 ± 9.60**	**130.77 ± 36.15**	**160.22 ± 42.09**	**4.62 ± 0.98**

Data are mean (standard deviation) unless otherwise indicated.

Significant values are in bold.

*BMI* body mass index, *HDL-C* high-density lipoprotein cholesterol, *LDL-C* low-density lipoprotein cholesterol, *TC* total cholesterol, *WHR* waist to hip ratio, *TG* triglyceride, *SBP* systolic blood pressure; *DBP* diastolic blood pressure, *FPG* fasting plasma glucose.

### Prevalence of dyslipidemia and other lipid panel abnormalities

**Figure 2:**
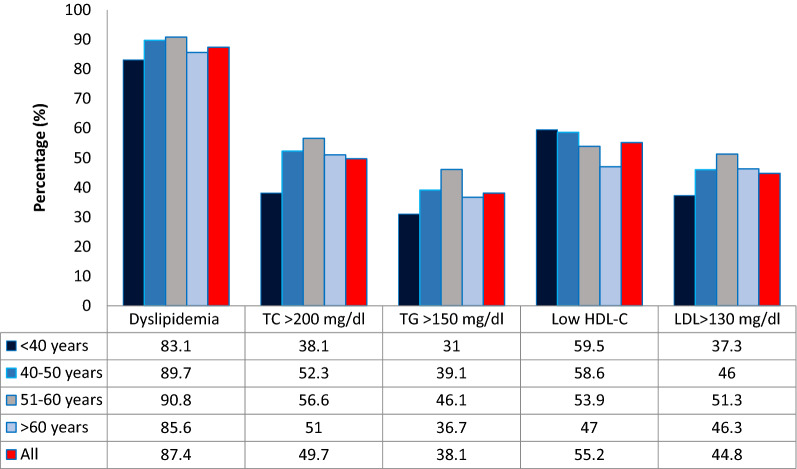
Prevalence of dyslipidemia within specific age strata.

At least one dyslipidemia was present in 87.4% (95% CI 84–90.8%) of the study respondents. The most prevalent lipid abnormality was low HDL-C (55.2%) followed by high TC [192 (49.7%)]; high LDL 173 [(44.8%)] and hypertriglyceridemia [147 (38.1%)]. As seen in Fig. 2, the prevalence of high TC, high TG, low HDL, and high LDL increased with age.

### Participants lipid profiles as per the ATP III, adult treatment panel III risk schema

These analyses revealed that women were disproportionately affected across all TC risk strata: borderline (71 (57.3%) in females vs. 53 (42.7) in males) and high risk (52 (76.5%) females vs. 16 (23.5%) in males). Similarly, the pattern was significant across non-HDL-C risk bands (borderline high (60 (62.6%) in females vs. 46 (37.4) in males), high risk (32 (66.7%) females vs. 16 (33.3%) in males) and very high (32 (88.9%) in females vs. 3 (11.1%) in males. The same pattern was observed across LDL-C and HDL-C risk categories. Mean values for TC, TG, LDL-C, HDL-C, non-HDL-C, and TC/HDL ratio are also presented. See Table 3 for additional information.

**Table 3:** Gender comparisons and NCEP ATP III risk categories.

Variable	Male No. (%)	Female No. (%)	*P* value	Total No. (%)
**TC**	197.40 ± 37.96	209.9 ± 49.32	0.005*	**205.24 ± 45.77**
Optimal	75 (38.7)	119 (61.3)	**0.027 (7.24)**	194 (50.3)
Borderline	53 (42.7)	71 (57.3)		124 (32.1)
High risk	16 (23.5)	52 (76.5)		68 (17.6)
**Non-HDL-C**	155.89 ± 34.62	162.79 ± 45.83	0.029*	160.22 ± 42.09
Optimal	34 (41.0)	49 (59.0)	**0.039 (10.10)**	83 (21.5)
Near-optimal	46 (37.4)	77 (62.6)		123 (31.9)
Borderline high	45 (42.9)	60 (57.1)		105 (27.2)
High	16 (33.3)	32 (66.7)		48 (12.4)
Very high	3 (11.1)	32 (88.9)		27 (7.0)
**TG**	153.5 ± 62.60	139.1 ± 59.92	0.095*	144.50 ± 61.26
Normal	79 (33.1)	160 (66.9)	0.088 (4.85)	239 (61.9)
Borderline High	37 (44.0)	47 (56.0)		84 (21.8)
High	28 (44.4)	35 (55.6)		63 (16.3)
**LDL-C**	125.35 ± 31.56	133.98 ± 38.31	0.018*	130.77 ± 35.15
Optimal	43 (50.6)	42 (49.4)	**0.003 (16.31)**	85 (22.0)
Near-optimal	38 (30.2)	88 (69.8)		126 (32.6)
Borderline High	44 (44.4)	55 (55.6)		99 (25.6)
High	14 (25.9)	40 (74.1)		54 (14.0)
Very high	5 (22.7)	17 (77.3)		22 (5.7)
**HDL-C**	41.50 ± 8.10	47.54 ± 9.72	0.001*	45.28 ± 9.60
Optimal	5 (15.2)	28 (84.8)	**0.001 (25.92)**	33 (8.5)
Borderline	75 (52.8)	67 (47.2)		142 (36.8)
High Risk	64 (30.3)	147 (69.7)		211 (54.7)

Significant values are in bold.

*LDL-C* low-density lipoprotein, *HDL-C* high-density lipoprotein, *TC* total cholesterol, *TG* triacylglycerol.

*t test.

### Prevalence of mixed dyslipidemias

As seen in Table 4, mixed dyslipidemia, defined as the presence of ≥ 2 lipid abnormalities, was also analyzed. Among all respondents, 59.6% (95% CI 54.6–64.6%) had mixed dyslipidemias. Most notably, respondents with abnormalities in two lipid variables presented either with elevated TG plus low HDL-C (39 (10.2%) or high TC plus high LDL-C, (51 (13.4%). High TC, TG, and LDL-C was the most common presentation (80 (20.9%) in respondents presenting with 3 lipid abnormalities. All four dyslipidemias occurred in 33 (8.6%) of the respondents. See Table 4 for further information.

**Table 4:** Mixed dyslipidemia.

Lipid sbnormality	Male N (%)	Female N (%)	Total N (%)
No Lipid abnormality	22 (45.8)	26 (54.2)	**48 (12.6)**
**Isolated dyslipidemia**			
*One abnormality*			
TC	1 (12.5)	7 (87.50)	8 (2.1)
TG	4 (80.1)	1 (20.0)	5 (1.3)
HDL-C	28 (31.1)	62 (68.9)	90 (23.6)
LDL-C	1 (33.3)	2 (66.7)	3 (0.3)
Total			**106 (27.3)**
**Mixed dyslipidemia**			
*Two abnormalities*			
TG + Low-HDL-C	18 (46.2)	21 (53.8)	39 (10.2)
LDL + Low-HL-C	1 (20.0)	4 (80.0)	5 (1.3)
TC + TG	7 (63.6)	4 (36.4)	12 (3.14)
TC + LDL-C	19 (37.3)	32 (62.7)	51 (13.4)
Total			**107 (28.04)**
*Three abnormalities*			
TG + TC + HDL-C	1 (12.5)	7 (87.5)	8 (2.1)
TC + TG + LDL	30 (6)	50 (94)	80 (20.9)
Total			**88 (23.0)**
*Four abnormalities*			
TG + TC + HDL-C + LDL-C	10 (30.3)	23 (62.8)	33 (8.6)
**Dyslipidemia**	**142 (37.2)**	**240 (62.8)**	**334 (87.4)**

Significant values are in bold.

*TG* triacylglycerol, *TC* total cholesterol, *HDL-C* high-density lipoprotein cholesterol, *LDL-C* low-density lipoprotein cholesterol.

## Logistic regression analysis of factors associated with lipid levels

### Factors associated with elevated non-HDL-C, TG, and TC

We summarize here the results of the multivariate models in Table 5. In this analysis, alcohol consumption (aOR = 2.24, 95% CI 1.34–3.74, *p* = 0.002); Family history of DM (aOR = 1.04, 95% CI 1.02–1.07, *p* = 0.001), FPG (aOR = 1.02 per 1 mg/dL, 95% CI 1.00–1.04, *p* = 0.008) had a strong independent association with abnormal non-HDL-C. In a separate analysis, likelihood of high TG was higher in females (aOR = 1.71, 95% CI 1.10–2.65, *p* = 0.008); alcohol consumers (aOR = 1.76, 95% CI 1.11–2.79, *p* = 0.014). Increasing WC (aOR = 1.03 per 1 cm, 95% CI 1.01–1.05, *p* = 0.002) was also associated with high TG. Although present in the adjusted model, the association between TG and family history of DM (aOR = 0.536, 95% CI 0.264–1.09, *p* = 0.086) or FPG (aOR = 1.01 per 1 mg/dL, 95% CI 0.998–1.02, *p* = 0.114) were not significant. Further, high TC was associated with alcohol consumption (aOR = 2.01, 95% CI 1.30–3.11, *p* = 0.002); presence of hypertension (aOR = 2.19, 95% CI 1.35–3.53, *p* = 0.001) and increasing concentration of FPG (aOR = 1.01 per 1 mg/dL, 95% CI 1.00–1.02, *p* = 0.001). See Table 6 for further information.

**Table 5:** Association between LDL, TC, TG, HDL, Non-HDL and TG/GDL ratio with key risk factors: result from logistic models.

Stratification variables	OR of low HDL mg/dL		OR of LDL ≥ 130 mg/dL		OR of TC/HDL > 5		Dyslipidemia	
	Crude OR (95% CI)	Adjusted OR (95% CI)	Crude OR (95% CI)	Adjusted OR (95% CI)	Crude OR (95% CI)	Adjusted OR (95% CI)	Crude OR 95%(CI)	Adjusted OR 95%(CI)
**Age (years)**	0.99 (0.67–1.01)	0.98 (0.97–1.00)	1.01 (0.99–1.03)		1.00 (0.97–1.02)		1.01 (0.98–1.04)	
**Sex**								
Female	1		1	1	1	**1**	1	
Male	0.824 (0.47–1.44)		0.696 (0.39–1.24)	0.68 (0.44–1.08)	1.86 (0.99–3.50)	**1.84 (1.00–3.38)**	0.76 (0.35–1.65)	
**Education**								
> High school	1		1		1		1	
< High school	1.14 (0.70–1.85)		0.76 (0.47–1.23)		0.74 (0.44–1.26)		0.89 (0.42–1.86)	
**Employment status**								
Unemployed	1	**1**	1		1		1	**1**
Employed	0.58 (0.32–1.03)	**0.51 (0.32–0.83)**	0.85 (0.47–1.52)		0.714 (0.37–1.37)		0.52 (0.22–1.23)	**0.48 (0.24–0.97)**
Self-employed	0.61 (0.30–1.25)	**0.52 (0.27–0.975)**	0.79 (0.38–1.63)		0.765 (0.35–1.68)		0.47 (0.17–1.30)	**0.41 (0.17–1.00)**
**Alcohol consumption**								
No	1	**1**	**1**	**1**	1		1	
Yes	0.42 (0.267–0.68)	**0.419 (0.265–0.66)**	**1.83 (1.16–2.90)**	**1.80 (1.16–2.81)**	1.17 (0.711–1.93)		0.96 (0.48–1.92)	
**Marital status**								
Not married	1		1	1	1	**1**	**1**	**1**
Married	1.32 (0.77–2.26)		1.64 (0.94–2.87)	1.56 (0.92–2.65)	1.90 (0.97–3.74)	**1.88 (1.00–3.63)**	**2.40 (1.18–4.89)**	**2.35 (1.19–4.66)**
**Family history of DM**								
No	1.45 (0.73–2.88)		1		1		1	
Yes			0.69 (0.354–1.34)		1.13 (0.56–2.27)		1.41 (0.50–3.97)	
**WC (cm)**	1.00 (0.97–1.02**)**		1.02 (0.997–1.05)	**1.03 (1.00–1.05)**	**1.04 (1.00–1.07)**	**1.03 (1.00–1.05)**	0.98 (0.93–1.03)	
**BMI (kg/m**^**2**^**)**								**1.09 (0.95–1.26)**
**< 25**	1	1	**1**	1	1			
**> 25**	0.63 (0.36–1.10)	0.65 (0.42–1.02)	**1.72 (0.99–3.01)**	1.70 (0.99–2.92)	1.27 (0.69–2.34)			
**Hypertension**								
No	1		1	**1**	1	**1**	1	
Yes	0.78 (0.42–1.47)		1.74 (0.933–3.25)	**2.06 (1.26–3.37)**	1.60 (0.83–3.10)	**1.72 (1.03–2.88)**	1.52 (0.54–4.29)	
**DBP (mmHg)**	1.01 (0.98–1.05)		1.01 (0.91–1.04)		1.03 (0.99–1.07)		1.05 (0.99–1.11)	**1.04 (1.00–1.09)**
**SBP (mmHg)**	0.99 (0.97–1.01)	0.99 (0.98–1.00)	1.01 (0.99–1.02)		1.00 (0.98–1.01)		0.89 (0.42–1.86)	
**FPG (mg/dL)**	1.98 (1.16–3.36)	**2.10 (1.25–3.52)**	1.00 (0.99–1.01)		**1.01 (1.00–1.023)**	**1.012 (1.00–1.022)**	1.02 (0.99–1.05)	**1.02 (1.00–1.05)**

Significant values are in bold.

*TG* triacylglycerol, *TC* total cholesterol, *HDL-C* high-density lipoprotein cholesterol, *LDL-C* low-density lipoprotein cholesterol.

**Table 6:** Association between LDL, TC, TG, HDL, Non-HDL and TG/GDL ratio with key risk factors: results from logistic models.

Stratification variables	OR of Non-HDL ≥ 130 mg/dL		OR of TG ≥ 150 mg/dL		OR of TC ≥ 200 mg/dL	
	Crude OR (95% CI)	Adjusted OR (95% CI)	Crude OR (95% CI)	Adjusted OR (95% CI)	Crude OR (95% CI)	Adjusted OR (95% CI)
**Age (years)**	1.01 (0.985–1.034)		0.99 (0.97–1.01)		1.01 (0.99–1.03)	
**Sex**						
Male	1		1	**1**	1	
Female	0.46 (0.23–0.92)		1.61 (0.91–2.88)	**1.71 (1.10–2.65)**	0.81 (0.46–1.42)	
**Education**						
> High School	1		1		1	
< High School	1.13 (0.625–2.04)		1.03 (0.63–1.68)		0.84 (0.52–1.36)	
**Employment status**						
Unemployed	1		**1**		1	
Employed	1.77 (0.86–3.65)		1.40 (0.77–2.54)		0.80 (0.45–1.43)	
Self-employed	1.55 (0.62–3.90)		1.00 (0.40–2.10)		0.77 (0.38–1.58)	
**Alcohol consumption**						
No	**1**	**1**	**1**	**1**	**1**	**1**
Yes	**2.22 (1.30–3.80)**	**2.24 (1.34–3.74)**	**1.73 (1.07–2.28)**	**1.76 (1.11–2.79)**	**2.05 (1.30–3.23)**	**2.04 (1.33–3.15)**
**Marital status**						
Not married	1		1		1	
Married	1.49 (0.79–2.82)		0.53 (0.26–1.06)		1.38 (0.80–2.37)	
**Family history of DM**						
No	1		1	1	1	
Yes	0.83 (0.39–1.77)		0.49 (0.23–1.01)	0.53 (0.262–1.06)	0.713 (0.37–1.37)	
WC (cm)	**1.06 (1.02–1.09)**	**1.04 (1.02–1.07)**	**1.04 (1.02–1.06)**	**1.04 (1.02–1.06)**	1.01 (0.98–1.04)	
**BMI (kg/m**^**2**^**)**						
< 25	1		1		1	
> 25	1.37 (0.70–2.69)		0.89 (0.51–1.55)		1.25 (0.72–2.16)	
**Hypertension**						
No	1		1		1	**1**
Yes	1.56 (0.685–3.56)		0.88 (0.46–1.66)		1.54 (0.83–2.87)	**2.19 (1.35–3.53)**
**DBP (mmHg)**	1.00 (0.961–1.04)		1.02 (0.99–1.06)		1.03 (0.99–1.06)	
**SBP (mmHg)**	0.995 (0.98–1.02)		1.00 (0.99–1.02)		1.00 (0.986–1.02)	
**FPG**	1.02 (0.998–1.04)	**1.02 (1.00–1.04)**	1.01 (0.998–1.02)	**1.01 (1.00–1.02)**	1.01 (0.996–1.02)	**1.01 (1.00–1.02)**

Significant values are in bold.

*TG* triacylglycerol, *TC* total cholesterol, *HDL-C* high-density lipoprotein cholesterol, *LDL-C* low-density lipoprotein cholesterol.

### Factors associated with abnormalities in HDL-C, LDL-C, TC/HDL-C, and dyslipidemia

Risk factors significantly associated with low HDL-C under adjusted multivariable analysis were employment (employed: aOR = 0.51, 95% CI 0.32–0.83, *p* = 0.001) (self-employed: aOR = 0.52, 95% CI 0.27–0.975, *p* = 0.034), alcohol consumption (yes: aOR = 0.419 95% CI (0.265–0.66, *p* = 0.034), and increasing FPG (aOR = 2.10, 95% CI 1.25–3.52, *p* = 0.001). Higher odd of high LDL-C was associated with alcohol consumption (yes: aOR 1.80, 95% CI 1.16–2.81, *p* = 0.001); increasing WC (aOR = 1.03 per 1 cm, 95% CI 1.00–1.05, *p* = 0.001) and presence of hypertension (aOR = 2.06, 95% CI 1.26–3.37, *p* = 0.001). Although BMI ≥ 25 kg/m^2^ was associated with abnormal LDL in the crude model, the relationship was attenuated in the adjusted model. Finally, presence of dyslipidemia (at least 1 lipid abnormality) was associated with employment status (employed: aOR = 0.48, 95% CI 0.24–0.97, *p* = 0.015) (self-employed: aOR = 0.41, 95% CI 0.17–1.00, *p* = 0.018); marital status (married: aOR = 2.35, 95% CI 1.19–4.66, *p* = 0.009); increasing DBP (aOR = 1.04 mmHg 95 CI 1.00–1.09) = 0.001) and increasing FPG (aOR = 1.02 per 1 mg/dL, 95% CI 1.00–1.05, *p* = 0.001). See Table 7 for additional information.

**Table 7:** Relationships between 10-year risk for cardiovascular risk (according to Framingham risk score) and selected variables.

Variables	Low	Moderate	High	Very high	*P* value (*χ*^2^)
**Waist circumference (cm)**					
Normal	38 (23)	52 (32.5)	16 (32.7)	3 (27.3)	0.127 (4.12)
Abnormal	128 (77)	108 (67.5)	33 (67.3)	8 (72.7)	
Mean WC in cm (± SD)	93 ± 11.5	93.3 ± 11.4	95.2 ± 8.8	98.6 ± 7.4	0.3^a^
**BMI (kg/m**^**2**^**)**					
Underweight	10 (6)	6 (3.8)	1 (2)	0 (0)	**0.009**
Normal	75 (45.2)	83 (51.9)	36 (73.5)	3 (27.3)	
Overweight	57 (34.3)	54 (33.8)	9 (18.4)	8 (72.7)	
Obese	24 (14.5)	17 (10.6)	3 (6.1)	0 (0)	
Mean ± SD	25.19 ± 4.44	24.76 ± 3.93	23.6 ± 3.2	25.5 ± 3.3	0.131^a^
**FPG (mg/dL)**					
< 100 mg/dL	151 (91)	134 (83.8)	36 (73.5)	8 (72.7)	**0.011 (8.98)**
> 100 mg/dL	15 (9)	26 (16.3)	13 (26.5)	3 (27.3)	
FPG (mean ± SD)	93.13 ± 23.38	97.17 ± 21.20	103.02 ± 27.30	97.45 ± 12.6	0.055^a^
**Educational level**					
No formal education	5 (3)	18 (11.3)	6 (12.2)	4 (36.4)	**0.005 (18.6)**
Primary	50 (30.1)	53 (33.1)	16 (32.7)	4 (36.4)	
Secondary	73 (44)	63 (38.8)	13 (26.5)	1 (9.1)	
Higher education	38 (22.9)	27 (17)	14 (28.6)	2 (18.2)	
**Marital status**					
Married	141 (85)	117 (73.1)	38 (77.6)	6 (54.5)	**<0.001 (36.3)**
Single	14 (8.4)	18 (11.3)	0 (0)	0 (0)	
Divorced/widowed	11 (6.6)	25 (15.6)	11 (22.4)	5 (45.5)	

Significant values are in bold.

*WC* waist circumference, *BMI* body mass index, *FPG* fasting plasma glucose.

^a^ANOVA.

### Framingham risk scores: magnitude and relationships

**Figure 3:**
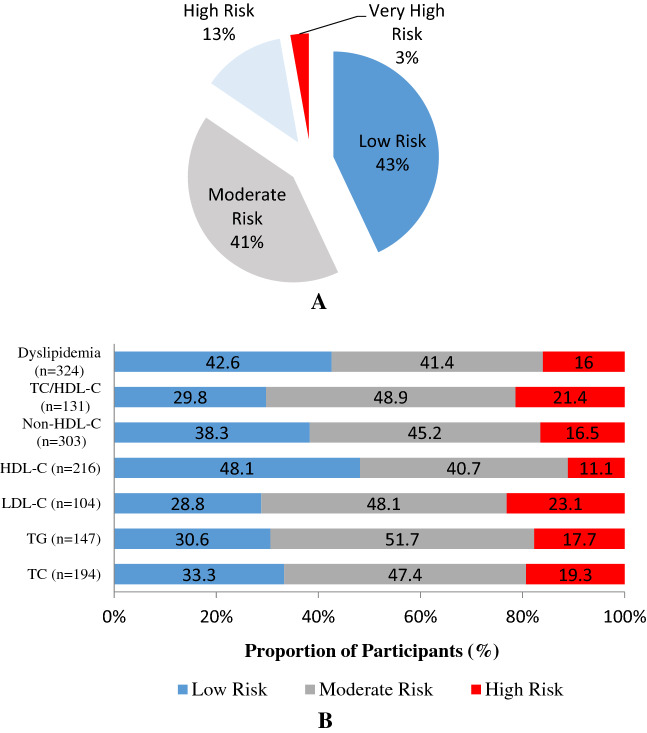
(**A**) Framingham risk score. (**B**) Relationship between specific lipid abnormalities and Framingham risk categories.

Ten years CVD risk scores were estimated using and Framingham CVD Risk Score Calculator. According to these estimates, 43% were at a low risk, 41.5% had a moderate risk; 12.7% had a high risk and 2.8% had a very high-risk of CVD events in the next 10-years. See Fig. 3A. Further, a separate analysis demonstrated that 42.6%, 41%, and 13% of patients with low risk, moderate risk and high risk of CVD events had at least 1 lipid abnormality (dyslipidemia). The dominant abnormalities in individuals in the high-risk category were high LDL-C and TC/HDL-C ratios. See Fig. 3B for additional associations.

## Discussion

Although more than 80% of the global burden of CVD is in LMIC, knowledge of important risk factors is largely based on extrapolations from HIC^4^. Furthermore, country-level analysis reveals important intra-country and inter-country differences in the combination of CVD risk factors (age, gender, tobacco smoking, diabetes mellitus (T2DM), lipid abnormalities/dyslipidemia, hypertension, obesity, and a family history of CVDs)^24,25^. For this reason, updated, context-specific data, on the burden or factors associated with CVD incidence, prevalence, morbidity, or mortality has been emphasized^4^. In this study, the first of its kind in Eritrea, we sought to evaluate the prevalence of dyslipidemias and its correlates among adults in Asmara, Eritrea. Moreover, 10-year CVD risk scores were estimated using and Framingham CVD Risk Score Calculator.

This study has many remarkable findings. Foremost is the fact that 87.4% of the study respondents had at least 1 lipid abnormality. Methodological differences and heterogeneity in cut-offs for relevant lipid markers notwithstanding; the estimate reported in this study is disproportionately high. However, comparable and, at times, higher estimates have been reported by some investigators in the region. For example, a study conducted in Nigeria reported a prevalence of 85.9%^6^. High values were reported in Lithuania (90%)^26^; Iran (83.4%)^27^, 85% in Kuwait^28^, South Africa (67.3%)^29^, United Arab Emirates (UAE) (72.5%)^30^, Makelle city, Northern Ethiopia (66.7%)^31^, India (ICMR-INDIAB study) (79%)^32^. A multi-country study (the Africa Middle East Cardiovascular Epidemiological (ACE) Study) reported a high prevalence of 70%^33^. However, a recent systematic and meta-analytical review estimated that the prevalence of dyslipidemia among adults in Africa is—15–50%^4,33^. In other words, the frequency of dyslipidemia in this population is higher than WHO estimates for Africa^34^ or what has been reported in some HICs^7^—52% in USA^35^.

Admittedly, the general term “dyslipidemia” can be misleading as it amalgamates disparate lipid components with varying, and at times debatable, contribution to CVD risk^36^. Therefore, the real-world consequences of the high and rising prevalence of dyslipidemia in populations in SSA are poorly understood. Indeed, the lack of prospective, well- controlled randomized trials that addresses the connection between specific CVD and low HDL-C has prevented definite conclusions at this point. Regardless, this study challenges the misconception that dyslipidemia is rare in SSA. Without a doubt, this presumption has created a false sense of security. In much of the continent, lipid health ranks low in the hierarchy of health priorities. Laboratory tests and anti-lipid medication are generally unavailable or unaffordable^4^. Altogether, the high prevalence of dyslipidemia in population across SSA highlights the need for commensurate health response—sustained surveillance of dyslipidemia and identification and implementation of context- specific interventions to address this problem.

Another important finding was the fact that the average values of TC, LDL, non-HDL-C; were above the cutoffs for abnormal values. Historically, multiple authors have argued that the mean values of lipid and lipoprotein biomarkers for populations in SSA are lower. For example, in the widely regarded INTERHEART study, the highest mean TC concentrations (> 200 mg/dL) were observed in Europeans and other Asians; intermediate levels (180–190 mg/dL) were observed for most of the regions, and the lowest means values (< 160 mg/dL) were observed in SSA^37^. In recent years, investigators have reported relatively high mean values for multiple lipid markers in the region^4^. Adeloye et al. reported a near-borderline population mean of ~ 189 mg/dL for TC^38^. In this study, women had significantly higher mean values of TC, HDL-C, and LDL-C. Across, disparate age bands, individuals in the 51–60 age bands had high mean values in TC, LDL-C, Non-HDL-C, and TC/HDL-C. Moreover, high mean values of multiple dyslipidemias were significantly associated with elevated WC, WHR, FPG, and alcohol consumption. While mean values of disparate lipid markers may reflect poorly on the burden of dyslipidemias in a population or a subgroup; the observed congruence between high averages in specific lipids markers and known cardiometabolic risk factors should raise concern. Therefore, the need for comprehensive measures to mitigate the health consequences is evident.

In terms of individual lipid and lipoprotein markers, the most predominant abnormality was low-HDL (55.2%), followed by hypercholesterolemia (49.7%), high LDL-C (44.8%), and hypertriglyceridemia (38.1%), respectively. Similar patterns have been reported in other settings in the region. For example, a study from Uganda reported that low HDL-C was the predominant (71.3%) lipid abnormality^37^. The prevalence of high TC, high LDL-C, and high TG were extremely low—6.0%), 5.2%, and 5.0%, respectively^34^. In a study conducted in South Africa (Soweto)^39^, the pattern among participants of African descent was as follows: low HDL-C (63%), high LDL-C (44%), high TC (39%), and high TG (23%). Recently, a prominent meta-analysis estimated that the frequency of low HDL-C was 37.4% (95% CI 29.4–45.7)^39^. Other dyslipidemias were in the following order: high LDL-C, 28.6% (95% CI 15.8–43.5) < total cholesterol, 25.5% (95% CI 20.0–31.4) < high TG 17.0%, 95% CI 11.9–22.7)^4^. With few exceptions^40,41^, low HDL-C is the most common lipid abnormality in SSA^4,7^. Reflecting on this observation, some investigators have suggested that HDL-C concentrations in Africa have declined gradually over the last 50 years^42^.

Although the high frequency of low-HDL in populations in SSA is well documented; the importance of this phenomenon is imperfectly understood. Part of the problem is the ambiguity concerning the relationship between HDL-C and CVDs. As previously noted, some investigators have concluded that low HDL-C concentration is not necessarily a marker of cardiometabolic risk in African populations^36,43^. Further, Mendelian randomization studies suggest that HDL-C is a CVD risk marker but not a true causal risk factor^43^. A phenomenon that has complicated this debate is the fact that low HDL-C coexists often with high TG or, like in our study, high LDL-C. In a study in Sweden, 37–38% had hypertriglyceridemia (150 and ≤ 354 mg/dL) with or without low HDL-C^44^. Therefore, dissecting the contribution of individual components (TG or HDL-C) to CVD risk is challenging. Even then, the observation that small, dense more atherogenic LDL-C (sdLDL-C) formation is inversely related to HDL-C concentration should raise concern.

Regarding causality, scholars have attributed the relatively low concentrations of HDL-C in populations across SSA to genetics^45^ or a range of modifiable risk factors including insulin resistance/type 2 diabetes mellitus (T2DM), BMI > 25 kg/m^2^, sedentarism/physical inactivity, overconsumption of carbohydrate, infection, and inflammation, among others^46,47^. In our study, we established a positive association between low HDL-C, elevated FPG, and employment status (predominance in the unemployed portion of the population). Similar to other studies^46,47^, consumption of alcohol was associated with increased concentration of HDL. In general, the mechanisms underpinning the positive correlation between alcohol consumption and HDL-C concentrations are not known. However, it has been hypothesized that alcohol may increase HDL-C by mediating the transport of apolipoprotein A1 (Apo-A1).

On the basis of currently available data, we believe that a large part of the observed outcome can be explained by physical inactivity and dietary factors. In general, Eritrea's traditional cuisine is starch- rich and emerging evidence supports the notion that sedentarism is a problem in Asmara^18^. Research in runners and the Framingham study demonstrated a strong inverse relationship between HDL-C concentration and physical activity ^48^. Likewise, carbohydrate over-nutrition can also lead to enhanced de novo lipogenesis and subsequent induction of ectopic lipid accumulation or aberrant lipid parameters. Unfortunately, individual-level data on nutrition was not documented in this study. Therefore, this explanation, although plausible, is speculative at best. Regardless, much work remains to be done on the relationship between low HDL-C and CVD or mechanisms behind the factors associated with low HDL-C. Addressing the genetics of HDL-C, diet, and exercise, or sedentarism should also be prioritized.

Beyond the debates on the connection between low-HDL-C and CVD; the nexus between TC, LDL-C, Non-HDL-C, and lipid ratios such as TC/HDL and CVD risk is unequivocally^9,33,48^. Genetical, observational, and interventional studies have established a connection between these abnormalities and CVD risk. The well-respected Multiple Risk Factor Intervention Trial (MRFIT) demonstrated a J-shaped curvilinear relationship between TC and CVD mortality^49^. Further, the evidence that LDL, particularly the smaller, denser more atherogenic (sdLDL) form, prospectively predicts hard CVD events (coronary death, myocardial infarction (MI), and stroke) is unequivocal^9^ and decreases generally correlate with improvement in clinical outcomes. Likewise, the INTERHEART Africa investigators concluded that TC/HDL-C and ApoB/apoA1 ratios provide equivalent information about CVD risk^8^. Importantly, abundant data suggest that non-HDL-C concentrations correlate very strongly with apoB and provide comparable clinical information. However, the nexus between TG (either fasting or non-fasting) concentrations and CVD is fraught with uncertainties and controversies^50^. On the whole, the high proportions and, to some extent, mean values of TC, LDL-C, TG, TC/HDL-C, and non-HDL-C observed in this setting should raise concern. Indeed, it’s our opinion that the results uncovered in this study may partially account for the high atherosclerotic cardiovascular disease (ASCVD)–related morbidity and general mortality rates observed in Eritrea.

To a large extent, most associations of TG, TC, TC/HDL-C, LDL-C, and Non-HDL-C were in the expected direction. High TC was independently associated with alcohol consumption, hypertension, and increasing FPG. The co-occurrence of high TC in combination with hypertension and elevated FPG is well documented in the region^51^. TC/HDL-C (one of the most potent predictors of CVD risk) exhibited an independent association with age, being married, elevated WC, presence of hypertension, and elevated FPG. The association between TC/HDL-C and known CVD risk markers appears to suggest that it can be a good marker of CVD risk in this population. Similar to other studies^49^, elevated LDL-C was associated with alcohol consumption, WC, and hypertension. These risk factors were also associated with non-HDL-C and TG (add sex) in this study. Remarkably, a large proportion of participants with elevated LDL-C were in the high-risk category in the 10-year Framingham CVD Risk estimates. Another interesting relationship was the observed association between TC/HDL-C ≥ 5 ratio and sex (higher in males); WC, hypertension, and FPG.

Despite the broad agreement between this study and other studies, notable exceptions were observed. For example, BMI ≥ 25 kg/m^2^ had only one association (LDL-C). This was in contrast to studies that have uncovered a significant relationship between elevated BMI, high TG, and low HDL-C^52^. We are unable to provide definitive explanations why BMI is a poor marker of dyslipidemia in this setting. However, this unexpected finding highlights the fact that the frequency of dyslipidemia can be high even in populations with relatively low prevalence of general obesity. Interestingly, we found a significant relationship between WC and multiple dyslipidemias in the multivariate analysis–Non-HDL-C, LDL-C, TC/HDL, and TG. As previously noted^53^, the use of WC for public health screening or clinical evaluation of patients is still limited in Eritrea. In this regard, the current study merely adds to the evidence of its utility and relevance in Eritrea.

Further, troubling associations and patterns were apparent in this population. The high number of women in NCEP ATP III high risk of very high-risk category; the higher likelihood of dyslipidemia in the unemployed; large number of individuals who are divorced/or widowed or without formal education in the high-risk category in Framingham 10-year general CVD risk estimate. The clustering of CVD risk markers among the unemployed, in populations of low socioeconomic status, or among the less educated strata of the society is well documented^46^. According to some authors, education mediates the risk of CVD through urbanization, unemployment, access to information/awareness, food, social support and cohesion, and individual health behaviors. The influence of these factors on CVD risk is poorly documented in populations across Eritrea.

By most accounts, mixed dyslipidaemia is both poorly described and inadequately addressed in current guidelines^9^. In this study, mixed dyslipidemia was relatively common (68.6%). For example, 28.04%, 23%, and 8.6% of the study participants had abnormalities in two, three, and four lipid components, respectively. The most common combination was high TC + TG + LDL-C (20.9%). The proportion of participants with high TC + low HDL-C and high TG + low HDL-C was also substantial—13.4% versus 10.2%, respectively. In general, the figures in our study are higher than those from Canada, Iran, and France^25,27,54^. This aside, we have to emphasize the fact that much of what we know about mixed dyslipidemia is based on studies from HIC. Therefore, rigorous prospective investigations are necessary to determine the risk associated with the simultaneous coexistence of specific lipid abnormalities in SSA. Nevertheless, there is little doubt that these combinations, by themselves, can accentuate CVD risk^25^. For example, epidemiologic studies suggest that the co-occurrence of high TG + low HDL-C concentrations (atherogenic dyslipidemia) is a strong risk factor for coronary heart disease (CHD) with post hoc analyses of several studies suggesting that these individuals have the highest rate of hard coronary events^53,55^.

### Strengths and limitations

To our knowledge, this is the first population-based study on the prevalence of elevated concentrations of TC, LDL-C, non-HDL-C, and TG and low HDL-C concentrations in adults in Eritrea. Regardless, this study is not without limitations. First, the cross-sectional nature of the study limits the dissection of causality. In addition, the fact that the population was mostly composed of urban residents limits the generalizability of our findings. The use of a researcher-administered questionnaire to capture data on specific variables may be affected by the recall, social desirability, and outcome misclassification biases. Lastly, the Framingham risk score and Friedewald equation for LDL-C estimation have not been validated in this population; hence the results should be used with caution. Despite the above limitations, and in the absence of longitudinal studies, this investigation represents a major first step towards getting baseline data on lipid profiles in Asmara, Eritrea. The assessment of all major lipoproteins and proportions of mixed dyslipidemias is rare in community-based studies in SSA and adds another layer to the information this paper provides on lipid abnormalities and CVD risk. Finally, the attempt to analyze 10-year general CVD risk adds to its uniqueness.

## Conclusion

This study uncovered many important findings. First, the prevalence of dyslipidemia is high in the general adult population in Asmara Eritrea. In reducing the frequency, the dominant abnormalities were: low HDL-C (55.2%), high TC (49.7%); high LDL (44.8%), and high TG. Analysis based on NCEP ATP III specifications demonstrated that women were disproportionately affected across all TC risk strata: borderline (71 (57.3%) in female vs 53 (42.7) in males) and high risk (52 (76.5%) females vs 16 (23.5%) in males). A similar pattern was observed across non-HDL-C, LDL-C, and HDL-C risk bands. Further, 59.6% had mixed with TC + TG + LDL-C combination predominating. Interestingly, multivariate logistic regression demonstrated that the presence of dyslipidemia was lower in individuals who were employed or self-employed; higher in those who were married; and was positively correlated with increasing DBP and increasing FPG. In terms of 10-year Framingham risk scores, 166 (43%), 160 (41.5%), 49 (12.7%), 11 (2.8%) were in the low-risk, moderate risk, high-risk, and very high-risk strata. As a whole, these unique data strongly suggest that dyslipidemia may be a principal contributor to CVD risk in this setting. The level of awareness is low and most study participants were not receiving lipid-lowering therapy as specified in international guidelines. Significantly, these observations call for concerted, effort directed at scaling up early recognition and treatment, including optimal pharmacological and non-pharmacological therapy at all levels of care. Lastly, further research is needed to corroborate our findings and to determine the ethnic-specific relationship between specific lipid markers or mixed dyslipidemias and CVD risk in this population.

## Data Availability

The dataset supporting the conclusions of this article are available from the corresponding author on reasonable request.
